# COVID-19 infection and return-to-play outcomes of elite athletes in Japan: a retrospective descriptive study

**DOI:** 10.1136/bmjsem-2025-002731

**Published:** 2025-10-27

**Authors:** Kazutaka Fukushima, Kazuyuki Kamahara, Anna Tomori, Yoshio Nakata

**Affiliations:** 1 Department of Sport Medicine, Japan Institute of Sports Sciences, Japan High Performance Sport Center, Tokyo, Japan; 2 Graduate School of Comprehensive Human Sciences, University of Tsukuba, Ibaraki, Japan; 3 Institute of Health and Sport Sciences, University of Tsukuba, Ibaraki, Japan

**Keywords:** COVID-19, Athlete, Epidemiology, Elite performance, Sports medicine

## Abstract

**Objectives:**

To describe the characteristics of COVID-19 among elite Japanese athletes and their return-to-play (RTP) time.

**Methods:**

We retrospectively reviewed clinical records at the Japan Institute of Sports Sciences between June 2022 and May 2023. Elite athletes who underwent periodic health examinations were examined by a physician to confirm COVID-19 history, symptoms and the RTP time.

**Results:**

Of 994 athletes, 456 had a COVID-19 history (mean±SD, 23.3±4.6 years; 56% male). Most infections occurred during the sixth wave (Omicron variant), followed by the seventh and eighth waves, with 88% recorded after the fifth wave. Indoor athletes were more frequently affected than outdoor athletes (306 vs 150, p<0.05). Badminton athletes were the most commonly affected athletes (16%), followed by volleyball (10%) and handball (7%). Among those with a history of COVID-19, 89% reported symptoms, while 11% were asymptomatic. Fever was the most common symptom (80%), followed by sore throat (58%) and cough (44%). The median (IQR) RTP time was 10 (7–14) days. Overall, 472 athletes resumed play within 28 days, while 20 returned after 28 days. RTP delays were more frequent before Omicron (9/59 athletes) than after (11/433 athletes, p<0.05).

**Conclusion:**

COVID-19 was more common among indoor sports athletes, primarily during the Omicron wave, with most cases being symptomatic but resolving quickly. These findings likely reflect factors such as close-contact training, immune changes during intensive training and international travel and may help characterise COVID-19 outcomes in elite athletes.

**WHAT IS ALREADY KNOWN ON THIS TOPIC**

- Athletes tend to be mildly affected by COVID-19, though some exhibit symptoms during exercise. Additionally, some infected athletes, particularly those experiencing chest pain, may require more than 28 days to return to play.

**WHAT THIS STUDY ADDS**

- This retrospective records review revealed COVID-19 was more frequent among indoor athletes, predominantly during the Omicron wave. Most cases were mild or asymptomatic, with 96% returning to play promptly and only 4% experiencing prolonged time loss (more than 28 days).

**HOW THIS STUDY MIGHT AFFECT RESEARCH PRACTICE OR POLICY**

- These results may be valuable for guiding COVID-19 clinical management and decisions regarding return to play.

## Introduction

 COVID-19 has substantially impacted athletes’ sports activities, with health effects extending beyond the early phase of infection. Some athletes reported experiencing symptoms during exercise postrecovery^1 2^ as well as an increased incidence of novel sport-related injuries on return to training.^3^ Emerging evidence also links pandemic-associated psychological stressors to dysregulation of extracellular signalling mechanisms, contributing to manifestations such as acute fatigue, depression and anxiety.^4^

Epidemiological data suggest that most athletes who contracted COVID-19 experienced mild symptoms with minimal impact on athletic performance, enabling a relatively swift return to play (RTP).^5^ However, the interpretation of these data may vary between elite and non-elite athletes. Elite athletes, often professionals, face intense pressure to continue competing and may experience significant consequences if they are unable to do so, regardless of symptom severity. They also have opportunities to participate in large-scale international competitions, and, in some cases, are required to undergo COVID-19 testing. Thus, the rate of positive results may potentially increase because of the frequent testing opportunities. Additionally, international travel, a known risk factor for disease transmission,^6^ further distinguishes elite athletes from non-elite counterparts. Consequently, the epidemiological trends of COVID-19 in elite athletes may differ from those seen in non-elite or recreational athletes. Nevertheless, evidence on the prevalence and impact of COVID-19 among elite athletes remains limited.

Owing to limited data-driven evidence, expert opinions remain the primary resource for determining the appropriate duration of interruption to practices and competitive activities.^5 7^ Therefore, in this study, we aimed to assess the frequency, distribution and RTP timeline associated with COVID-19, in order to describe the characteristics of elite athletes with COVID-19 in Japan. These results may inform clinical management and RTP protocols for elite athletes in future pandemics.

## Methods

### Study design and participants

This retrospective descriptive study was conducted in accordance with the Strengthening the Reporting of Observational Studies in Epidemiology guidelines.^8^ Data were collected from the clinical database of the sports clinic at the Japan Institute of Sport Sciences (JISS) by reviewing records on COVID-19 history and information about elite athletes who underwent periodic health examinations (PHEs). The analysis was conducted between October 2023 and March 2024. Medical records included information about elite athletes who underwent PHEs between June 2022 and May 2023, when JISS began conducting interviews regarding COVID-19. JISS is a central facility for sports medicine and science in Japan, equipped with various laboratory and training facilities, as well as a dedicated practice field for each sport. Participants were elite athletes permitted by the Japanese Olympic Committee, the Japanese Paralympic Committee or their affiliated athletic organisations to use the JISS facilities to improve their international competitiveness. No special exclusion criteria were established.

Comprehensive consent for the use of medical treatment data in research was obtained in advance, and an opt-out procedure was implemented for the release of research information and for declining participation owing to non-consent. During PHEs, a physician interviewed each athlete regarding their COVID-19 history following a prescribed form. The questions included age, sex, date of illness, reason for testing, route of infection, symptoms (most typical of COVID-19)^5 9^ and time to RTP. Furthermore, the athletes reported the number of days to RTP when asked, ‘How many days did it take you to return to regular practice after you tested positive for COVID-19?’ This was based on one variant of the definition of RTP: “return to practice or training.”^10^

### General population comparison

To describe the change in the number of new cases over the study period, a bar graph was created to compare elite athlete data with that of the general Japanese population in their 20 s.^11^ These data, compiled by the Ministry of Health, Labour and Welfare based on the Health Center Real-time Information-sharing System on COVID-19 data, include the number of new COVID-19 cases in their 20s between September 2020 and May 2023. Additionally, the athletes’ infection period (August 2020–March 2023) was categorised into the following eight epidemic waves: first (13 January 2020–7 June 2020), second (8 June 2020–27 September 2020), third (28 September 2020–28 February 2021), fourth (1 March 2021–20 June 2021), fifth (21 June 2021–28 November 2021), sixth (29 November 2021–19 June 2022), seventh (20 June 2022–9 October 2022) and eighth (10 October 2022–31 March 2023). Each epidemic wave was defined by a start week when the number of cases ‘increased for at least 3 weeks and was ≥10% of the peak or ≥1.5 times the peaks for 2 consecutive weeks’, and an end week, when cases ‘decreased for at least 3 weeks and fell to ≤10% of the peak (before the next wave started)’. The dominant strains for each wave were B lineage (third wave), Alpha (fourth wave), Delta (fifth wave), Omicron BA.1/BA.2 lineage (sixth wave), Omicron BA.5 lineage (seventh wave) and Omicron XBB lineage (eighth wave).^12^

### Equity, diversity and inclusion statement

Elite Japanese athletes who underwent PHEs at JISS were recruited for this study. Participants represented diverse backgrounds, including teams and individuals, summer and winter sports and Olympic and Paralympic sports. Both male and female athletes were included, as indicated in the results. The research team comprised three male and one female Japanese researchers, alongside research scientists, a senior researcher and a professor with expertise across various fields, including respiratory medicine, paediatrics, emergency medicine and sports medicine.

### Patient and public involvement

Athletes were not engaged in the development, conduct, or oversight of the study.

### Statistical analysis

Descriptive statistics (frequency, median, SD and crosstabs) were used to summarise data. Categorical variables were presented as numbers and percentages. χ^2^ or Fisher’s exact tests were applied to compare distributions of categorical variables. Data normality was assessed using histograms. Age, which was normally distributed, was reported as mean±SD and compared using the Student’s t-test or Welch’s test. Time to RTP, which was non-normally distributed, was reported as the median with IQR. For all statistical analyses, a two-tailed p<0.05 was considered statistically significant. Data were analysed using IBM SPSS Statistics, V.29.0.2.0 (IBM, Armonk, New York).

## Results

### Characteristics of athletes with or without a previous medical history of COVID-19

Table 1 shows the characteristics of athletes with and without a previous medical history of COVID-19. In total, 994 athletes (517 males and 477 females) underwent PHE during the study period. The mean age±SD of the total athletes was 22.9±4.8 years. Among them, 456 athletes had a history of COVID-19, with a total of 492 documented COVID-19 cases. Of these, 422 players had been infected once, 32 twice and 2 three times. Regarding sports places, indoor sports had significantly more athletes with a history of COVID-19 compared with that of outdoor sports. The highest proportions were observed in badminton (16%), volleyball (10%) and handball (7%). Among those with a history of COVID-19, 89% reported some symptoms, whereas 11% were asymptomatic. Around the sixth wave, when the Omicron strain became dominant, the most common symptoms were fever, fatigue, sore throat and cough. Nearly half the athletes who contracted the virus before the emergence of the Omicron variant reported anosmia and dysgeusia; however, this prevalence decreased to around 10% after the spread of Omicron (online supplemental table 1).

**Table 1:** Characteristics of athletes with and without a past medical history of COVID-19 in Japan between 2022 and 2023

Characteristics		Total athletes n=994 n (%)	Athletes with a history of COVID-19 n=456 n (%)	Athletes without a history of COVID-19 n=538 n (%)	P value*
Age (mean±SD)		22.9±4.8	23.3±4.6	22.7±5.0	0.032
Sex	Male	517 (52)	254 (56)	263 (49)	0.032
Sport category					0.242
	Summer sport	759 (76)	356 (78)	403 (75)	
	Winter sport	235 (24)	100 (22)	135 (25)	
Sports place					<0.001
	Indoor	575 (58)	306 (67)	269 (50)	
	Outdoor	419 (42)	150 (33)	269 (50)	
Sports type					
	Badminton	93 (9)	71 (16)	23 (4)	
	Volleyball	82 (8)	49 (10)	35 (7)	
	Handball	52 (5)	32 (7)	20 (4)	
	Swimming	75 (8)	31 (7)	44 (8)	
	Ice hockey	48 (5)	27 (6)	21 (4)	
Symptoms					
	Asymptomatic		50 (11)		
	Symptomatic		406 (89)		
	Fever		365 (80)		
	Sore throat		266 (58)		
	Cough		202 (44)		
	Fatigue		139 (30)		
	Headache		105 (23)		

* P value: Student’s t-test was used for age, χ2 tests or Fisher’s exact test were used for other categorical variables. p<0.05 indicates statistical significance.

### Reasons for coronavirus disease infection testing

The primary reasons for COVID-19 testing included being symptomatic (75%), close contact with an infected person (14%), travel requirements (5%), facility use (3%), and other reasons (3%), such as regular team check-ins for negative certificate and screening owing to a teammate testing positive, despite no close contact.

### COVID-19 case trends among athletes and the general Japanese population by epidemic wave

**Figure 1:**
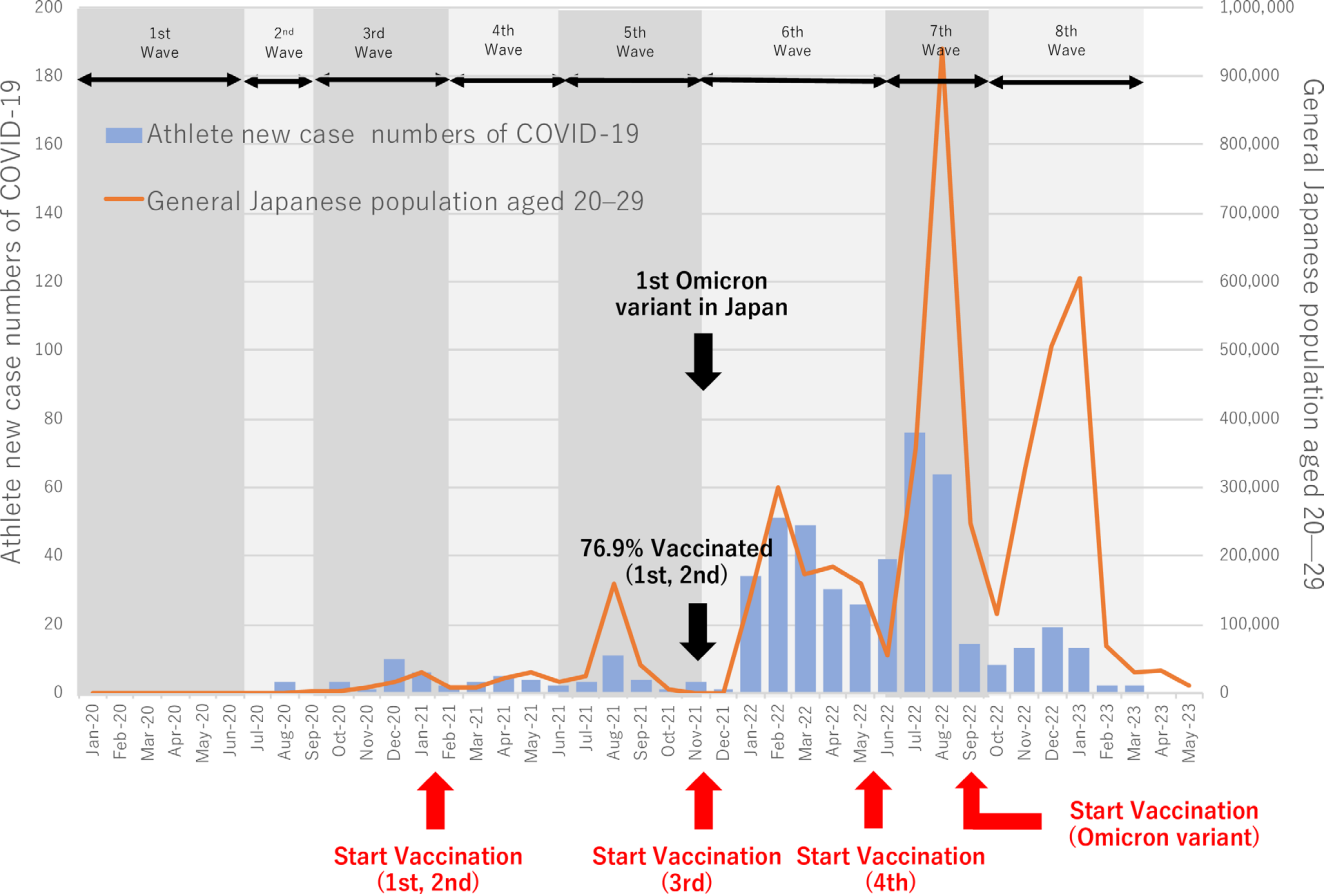
Number of new cases of athletes and general Japanese population aged 20–29 with COVID-19.

Monthly COVID-19 cases among athletes are shown in figure 1, with cases in the general Japanese population aged 20–29 depicted as a line graph. Cases by epidemic wave in both groups are summarised in online supplemental table 2. Among athletes, the sixth wave accounted for the largest number of infections (224 cases), while the eighth wave included 49 cases. In the general population, the sixth wave accounted for 5 21 401 cases, while the eighth wave was the largest, comprising 1 602 554 cases.

### Return-to-play

**Figure 2:**
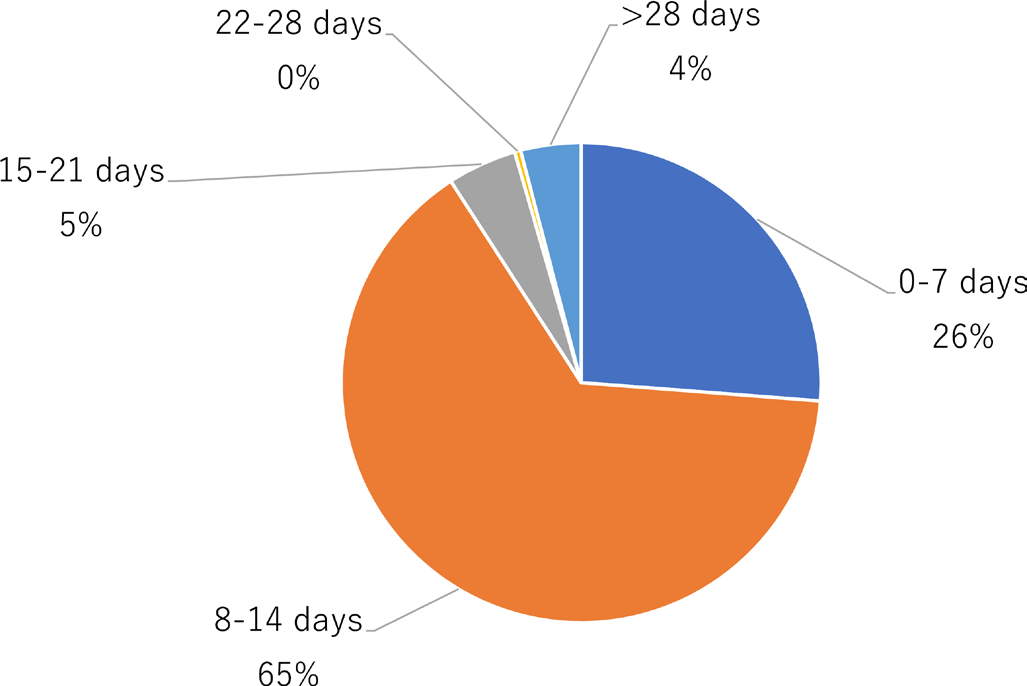
Number of days from the onset of COVID-19 to RTP. Data are presented in %. RTP, return to play.

RTP within 0–7 and 8–14 days accounted for 90% of cases (figure 2). The median (IQR) time to RTP was 10 (7–14) days. Overall, 20 (4%) players required >28 days to return, all of whom were symptomatic. The most common symptom was fever, followed by cough, fatigue and sore throat (table 2). When comparing the period before the sixth wave (first to fifth waves), prior to the dominance of the Omicron strain,^12^ with the period after the sixth wave (sixth to eighth waves), the proportion of RTP >28 days was 15% (9/59 athletes) and 3% (11/433 athletes), respectively (p<0.05). Notably, 88% of all COVID-19 cases occurred after the fifth wave.

**Table 2:** Characteristics, sports category/type and symptoms associated with prolonged RTP time (>28 days)

Characteristics		RTP≤28 days n=472 n (%)	RTP>28 days n=20 n (%)	P value*
Age (mean±SD)		23.2±4.3	25.8±7.6	0.150
Sex	Male	256 (54)	13 (65)	0.344
Sport category				<0.001
	Summer sport	380 (81)	5 (25)	
	Winter sport	92 (19)	15 (75)	
Sports place				0.026
	Indoor	344 (73)	10 (50)	
	Outdoor	128 (27)	10 (50)	
Sports type				
	Volleyball	50 (11)	2 (10)	
	Ice hockey	30 (6)	2 (10)	
	Snowboard	11 (2)	2 (10)	
	Wheelchair basketball	10 (2)	2 (10)	
	Para alpine skiing	3 (1)	2 (10)	
	Handball	33 (7)	1 (5)	
	Swimming	32 (7)	1 (5)	
	Athletics	12 (3)	1 (5)	
	Table tennis	10 (2)	1 (5)	
	Ski jumping	7 (1)	1 (5)	
	Cricket	6 (1)	1 (5)	
	Shooting	4 (1)	1 (5)	
	Triathlon	4 (1)	1 (5)	
	Other	260 (55)	2 (10)	
Symptoms				
	Asymptomatic	55 (12)	0 (0)	
	Symptomatic	345 (73)	20 (100)	
	Fever	187 (40)	17 (85)	
	Cough	126 (27)	13 (65)	
	Fatigue	257 (54)	11 (55)	
	Sore throat	68 (14)	9 (45)	
	Anosmia/dysgeusia	100 (21)	8 (40)	
	Headache	93 (20)	5 (25)	
	Nasal discharge	6 (1)	2 (10)	
	Nausea	5 (1)	1 (5)	
	Diarrhoea	5 (1)	1 (5)	
	Other	58 (12)	0 (0)	

* P value: Welch test was used for age, while χ2 tests or Fisher’s exact test were used for other categorical variables. p<0.05 indicates statistical significance.

RTP, return to play.

## Discussion

In the current study, we identified the basic characteristics of elite athletes affected by COVID-19, including age, sex, sports discipline and clinical characteristics. COVID-19 was more prevalent among athletes participating in indoor sports, with the majority of infections occurring from the Omicron wave onward. While most cases were mild or asymptomatic, athletes returned to competition within 28 days and recovery times were shorter during the Omicron period compared with those of the earlier variants.

### Sports type and category

Our findings indicate that athletes engaged in indoor sports were more frequently affected by COVID-19 than those participating in outdoor sports, with particularly high case numbers in badminton, volleyball, handball, swimming and ice hockey. This trend aligns with previous reports from the Netherlands^13^ and the USA,^14^ which also highlighted an elevated risk of infection among athletes involved in indoor activities. The present study could not determine the exact timing or circumstances of infection; however, the observed pattern may be explained by the characteristics of indoor environments, where limited ventilation, close physical contact and aerosol transmission could increase susceptibility.

### Symptoms

Only 11% of infected athletes were asymptomatic. This contrasts with a study from Qatar, which reported 58.3% asymptomatic cases (21 of 36 test-positive individuals),^15^ and another study reporting 16%.^3^ Such disparities may be attributable to factors such as differences in the main COVID-19 variant^16^ and vaccination status.^17^ Vaccinations were recommended and initiated during the survey period. Among symptomatic athletes, the most commonly reported symptoms were systemic general (fever and fatigue) and respiratory (sore throat, cough and nasal discharge), while gastrointestinal symptoms (abdominal pain, nausea and diarrhoea) were less frequent. We examined symptoms before and after the Omicron strain became dominant and found that the above trends were the same.

A study from Hungary, involving elite and recreational athletes, reported that approximately 40% of patients experienced fatigue and palpitations.^3^ Similarly, data from elite athletes in the United Kingdom (UK) showed that approximately half reported fatigue, cough and headache.^5^ According to statistics from the general Japanese population,^18^ 2.1% were asymptomatic, while 62.7% reported cough, 60.7% sore throat, 44.3% nasal discharge, 42.1% headache and 38.8% fever. While these results showed some variation resulting from different viral variants^19^ and vaccination status,^17^ they likely reflect the same trends, indicating that systemic and respiratory symptoms were the primary manifestations, while gastrointestinal symptoms were auxiliary.

### COVID-19 infection curve

The trend in new COVID-19 cases among elite athletes was similar to that of the general Japanese population in their 20s. However, elite athletes were more susceptible to infection during the sixth wave than during the eighth wave, whereas the general population showed higher susceptibility in the eighth wave than in the sixth wave. During the sixth wave, when the primary strain was the Omicron variant (BA.1/BA.2),^20^ characterised by enhanced immune escape and increased transmissibility,^20^,^22^ the results may be explained by the higher risk in the athlete group who practise and compete with others at close distances. Moreover, the fact that over half of infected individuals can transmit the virus before exhibiting symptoms^23^ and the difficulty of diagnosing infection using qualitative antigen tests during the presymptomatic period^24^ might affect athletes in close-contact settings.

To prevent the spread of infection during the eighth wave, prefectural governments issued a new system that allowed measures to be taken without imposing legal penalties. This was in contrast with the previous government-issued ‘Declaration of a State of Emergency in Response to COVID-19’ and ‘Priority Measures to Prevent the Spread of Infections’, which involved penalties for violators. These changes have led to increased social activity and contact with others, resulting in a higher number of new positive cases among the general population during the eighth wave. In contrast, it can be argued that such an increase had minimal effect on the group of athletes.

### Reasons for testing for COVID-19 infection

Overall, 75% of athletes were tested owing to symptoms, while the remaining individuals tested positive incidentally through close contact with infected persons, travel requirements or facility use. Conversely, a study of college student athletes in the USA^25^ reported that 49.7% were tested through screening, 21.4% owing to symptoms and 6.9% via contact tracing. These differences in the percentage of symptomatic athletes between the current and previous studies may be attributed to the testing context: in the previous study, tests were performed before sports activity, which means that most symptomatic athletes would not have been included. Additionally, 5% of athletes in the present study were tested while travelling during periods of movement restrictions and were all asymptomatic. Given that they had been training as usual, it can be inferred that a risk of infection spread remained, even if thorough infection control measures were observed outside competitions, since wearing masks and maintaining a safe distance during training was difficult.

### Return-to-play

The median (IQR) time to RTP was 10 (7–14) days, with 65% of athletes returning within 8–14 days, 26% within 0–7 days and 5% within 15–21 days. This RTP duration was shorter than that reported in a previous study of British elite athletes (18 days (12–30); 27% took >28 days). However, it may have been influenced by the government’s length of isolation for patients who test positive for COVID-19. While the UK mandated 14 days of self-isolation before December 2020 and 10 days thereafter for symptomatic patients, Japan gradually loosened the isolation period from 14 to 7 days during the study period. Consequently, most Japanese athletes, who were forced to quarantine and then resume training immediately after its completion, would have had 10 days. Nevertheless, it should be noted that this RTP represents a generalised estimate influenced by both national isolation and the actual ‘time off’ from the sport period.

Twenty (4%) athletes required >28 days to RTP, a considerably smaller number compared with 27% reported in a previous study.^5^ The report that the Omicron variant causes ‘long COVID-19 syndrome’ with shorter periods than the Delta variant^26^ may have contributed to the lower number of athletes who required >28 days to resume sports activity in this study. As shown in the results, RTP >28 was significantly more common among athletes infected before the sixth wave, which was dominated by the Omicron strain, whereas most athletes surveyed in this study were infected after the sixth wave.

In a previous study, only chest pain at presentation was shown to be associated with longer time loss (>28 days)^5^; however, in the present study, chest pain was not reported in the prolonged RTP group (only one athlete had chest pain in the short RTP group).

### Strengths and limitations

#### Strengths

The current study successfully reported the distribution, characteristics and changes in elite athletes in a large sample over time. A key strength of this study is the inclusion of only elite athletes, representing over 50 different sports disciplines, and the same method was implemented in a single facility.

#### Limitations

A key limitation of this study is the reliance on participants’ self-reported information, which introduces the potential for recall bias, as individuals may not accurately remember or report past exposures. Such bias could affect the validity of our findings by either underestimating or overestimating associations. Future research could mitigate this issue through prospective data collection and the use of objective measurement techniques. Variations in the period between onset and questioning may also affect the accuracy of responses. Additionally, potential confounders, such as pre-existing diseases and vaccination status, were not identified in this study. The absence of symptom criteria may have led athletes to respond incorrectly when directly questioned by doctors about information regarding symptoms.

### Clinical implications

The characteristics of athletes who contracted COVID-19 may be influenced by the unique environment of elite athletes. Factors that significantly impact the risk of disease, including COVID-19, should be examined in future studies. Additionally, while the findings of this study suggest the difficulty of suppressing COVID-19 spread, they also point to the possibility of effective clinical management and a return to competition with minimal time loss.

## Conclusions

COVID-19 was more common among indoor sports athletes, particularly during the Omicron wave. Most cases were mild or asymptomatic, with athletes returning to competition within 28 days and experiencing shorter recovery times than those associated with earlier variants. Despite the limitations of self-reported data and unmeasured confounders, such as comorbidities and vaccination status, the present study highlights the resilience of elite athletes and underscores the need for context-specific infection control, monitoring and return-to-play strategies. Future research should integrate prospective designs and detailed clinical parameters to better elucidate risk factors, long-term outcomes and tailored interventions for this unique population.

### Recommendations

Some athletes are expected to continue being affected by COVID-19, and healthcare providers must ensure their safe RTP. In addition to assessing the risk of COVID-19 transmission, the impact of a period of exercise restriction, sequelae and risk of trauma after the resumption of exercise must be considered for evaluating the timing for RTP. ^27^

## Supplementary material

10.1136/bmjsem-2025-002731 online supplemental file 1

## Data Availability

Data are available upon reasonable request.
