# Supplementary material for: COVID-19 infection and return-to-play outcomes of elite athletes in Japan: a retrospective descriptive study
Source: BMJ Open Sport Exerc Med. 2025 Oct 27;11(4):e002731. doi: 10.1136/bmjsem-2025-002731 (PMC12574401; doi:10.1136/bmjsem-2025-002731)
Supplement: online supplemental file 1 [file bmjsem-11-4-s001.docx]

**Supplemental Table 1.**

|  | **Before the Omicron strain**  **n = 59**  **n (%)** | | **After the Omicron strain**  **n = 433**  **n (%)** |
| --- | --- | --- | --- |
| Fever | 36 (61) | 315 (73) | |
| Anosmia/dysgeusia | 29 (49) | 48 (11) | |
| Sore throat | 21 (36) | 239 (55) | |
| Cough | 16 (27) | 179 (41) | |
| Fatigue | 11 (19) | 121 (28) | |
| Headache | 8 (14) | 95 (22) | |
| Nasal discharge | 8 (14) | 86 (20) | |
| Diarrhoea | 1 (2) | 5 (1) | |
| Nausea | 0 (0) | 7 (2) | |
| Other | 7 (12) | 51 (12) | |
| Asymptomatic | 5 (8) | 50 (12) | |

※As each player experienced multiple symptoms, the total number of cases does not correspond to the total number of symptoms.

**Supplemental Table 2.**

| **COVID-19 wave** | **No. of athletes infected, n = 492** | **No. of the general population in their 20s infected,**  **n = 3,183,541** |
| --- | --- | --- |
| First | 0 | N/A |
| Second | 3 | 1,476 |
| Third | 21 | 35,522 |
| Fourth | 13 | 40,130 |
| Fifth | 22 | 129,120 |
| Sixth | 224 | 521,401 |
| Seventh | 160 | 853,338 |
| Eighth | 49 | 1,602,554 |
|  |  |  |

* N/A means “not available.” COVID-19, coronavirus disease
